# A Systematic Review of the Literature and Meta-Analysis of Autologous Fat Transfer: Fat Transfer Confers a 4.2% Incidence of Complications

**DOI:** 10.7759/cureus.97063

**Published:** 2025-11-17

**Authors:** Janak Bechar, Thomas Kidd, Howard Chu, Joseph Hardwicke

**Affiliations:** 1 Plastic Surgery, University Hospitals Coventry and Warwickshire, Coventry, GBR; 2 Plastic Surgery, University Hospitals Birmingham, Birmingham, GBR; 3 Plastic and Reconstructive Surgery, University Hospitals Coventry and Warwickshire, Coventry, GBR

**Keywords:** autologous fat transfer, facial reconstruction, fat emulsion, fat graft survival, fat survival

## Abstract

Reporting of complications such as infection, necrosis, oil cysts and haematoma after fat transfer varies widely in the literature. Numerous variations in techniques (for example, cannula size, infiltration solution and fat-processing methods) have been described across a heterogeneous patient population for age, fat harvest location and recipient site. To date, no comprehensive review of the literature has been performed for all fat transfer procedures across the body. This study aims to provide novel information on the incidence of complications after fat transfer and is unique in its size and scope.

A systematic review of English literature was performed from 01/01/08 to 01/09/24 in MEDLINE, EMBASE, PUBMED and Cochrane Database of Systematic Reviews. Measured complication outcomes included necrosis, infection, induration, oil cysts, haematoma and pneumothorax. A random-effects model was employed to calculate the pooled complication incidence from selected articles.

After application of inclusion and exclusion criteria, 42 articles progressed to final analysis with a total of 6268 patients. Major complications investigated were fat necrosis (n=44, 0.7%), infection (n=66, 1%), induration and calcification (n=40, 0.6%), oil cysts (n=7, 0.1%) and haematoma (n=4, 0.06%). Pneumothorax post-procedure incidence was 0.1% (n=6). Overall complication incidence was 4.2%.

This study is unique in its scope and size and can be used for counselling patients in the pre-operative setting, providing a benchmark for surgeons to assess their practice with an overall complication incidence of 4.2%.

## Introduction and background

First described by Neuber G [1] in 1893, fat transfer, or autologous fat grafting, has been used in a variety of applications, including correcting breast contour defects after cancer surgery and lower limb defects after major trauma. Fat transfer is common, constituting 5.9% of all surgical aesthetic procedures, with more than 514,000 procedures performed globally in 2009 [2,3]. Over 70% of plastic surgeons use fat transfer to reconstruct breast defects [2,3]. Various techniques of fat harvest and transfer have been described, the most common of which is the Coleman fat grafting technique [3]. 

Ideal areas for fat harvest have yet to be defined [4]; a donor site is usually the anterior abdominal wall or thigh. Harvesting involves the aspiration of a fat solution from a given donor site through a metal cannula. The aspirate contains both adipocytes and adipocyte-derived stem cells, which increase regenerative potential and vascularisation of the graft [5-7].

Introduction of too much fat can lead to increased interstitial pressure and a collapse of capillaries, leading to hypoxia and graft loss [3,8,9]. However, the success of fat graft take is highly variable. A systematic review of 16 clinical studies revealed that fat survival can be as little as 15% from six months to 3.7 years of follow-up [10]. 

Factors affecting complications after fat transfer are poorly described in the literature. Early complications (<4 weeks from the time of procedure) may include local surgical site infection (0.7% donor or recipient site not requiring surgery [11]) and major complications such as pulmonary embolism. Major complications needing surgical intervention or hospitalisation have been described in a limited manner (incidence of ~6% in breast patients [11]), with the incidence of infections requiring surgery in breast patients being 2% [11]. Late complications (>4 weeks from procedure) may include graft necrosis, resorption and calcification [12]. Low-morbidity complications are the most numerous at 60% (such as swelling and induration) after breast fat transfer [12]. The incidence of fat necrosis ranges from 3% to 17% [13-16] and is hypothesised to be influenced by high fat-transfer volume [14] and operative technique. 

By pooling the complications of worldwide studies, the incidence of specific complications can be elucidated for a variety of techniques. To date, no comprehensive review of the literature has been performed for all fat transfer procedures across the body. This study aims to provide novel information on the incidence of complications after fat transfer and is unique in its size and scope, and will thus provide a benchmark for patient outcomes in surgical practice.

## Review

Methods

*Data Sources* 

A systematic review of literature in English was performed using the following databases: MEDLINE, EMBASE, PUBMED, Cochrane Database of Systematic Reviews. The following keywords were used: [(Autologous fat grafting) OR (autologous fat transfer)] AND (complications). The publication date range was 01/01/2008-01/09/2024 to include more recent literature and practice. 

Study Selection

Each article was independently reviewed by two researchers (JAB and HC) for review. Eligibility criteria were defined using the population, intervention, comparator, outcome and study design approach (PICOS) [17]. Inclusion and exclusion criteria are summarised in Table 1. A subgroup of articles was included in a given paper if the data could be extracted and satisfied the inclusion and exclusion criteria.

**Table 1:** Inclusion and exclusion criteria applied to the literature search

	Inclusion criteria	Exclusion criteria	Data extracted	
				Population	Human	Non-human	Age at operation	
Cosmetic, oncology and trauma patients	Review articles; abstracts; conference proceedings; non-English language literature	Country of origin		
Over 18 years				
Any country of origin				
Reported as a full article in an English-language journal				
Intervention	Patients undergoing fat transfer	Patients undergoing liposuction exclusively	Surgical technique	
	Single-stage grafting	Multiple-stage grafting		
	Study cohort ³10 patients	Study cohort <10 patients		
Comparator	Coleman vs. other techniques	None	Comparison group	
	Donor/recipient sites			
Outcome	Complications	No complications recorded	Acute and delayed complications	
		Non-extractable data		
Study design	Any clinical study design (randomised or non-randomised; comparative or non-comparative)	Non-clinical study	Study design; method of randomisation; years of study; length of follow-up	

Article selection was determined by a two-level screening process. The first level consisted of abstracts being reviewed for the inclusion and exclusion criteria. The second level consisted of the criteria being applied to the entirety of the article and data being extracted. Data were included from a given paper if both levels of inclusion and exclusion were passed. The final list of articles was reached via consensus of both researchers to ensure the inclusion and exclusion criteria were met. Article evaluation followed the guidance of the Preferred Reporting Items for Systematic Reviews and Meta-analyses (PRISMA) criteria [17-19]. 

The Methodological Index for Non-Randomized Studies (MINORS) instrument was used to assess the methodological quality of non-randomised studies [20]. Randomised control studies (RCTs) were evaluated for methodological quality using the Detsky score [21-23]. Appraisal for each non-randomised study yielded a score from a maximum of 16 for non-comparative studies and 24 for comparative studies, with a maximum score of 20 for randomised studies. MINORS or Detsky score of at least 75% of the maximum was deemed to be of high quality [21,22]. 

*Data Extraction and Analysis* 

Extracted data were recorded using Microsoft Excel (Microsoft Corp., Redmond, WA, USA). The agreement between researchers selecting papers was described using a kappa statistic. A proportion meta-analysis was used to describe complications after fat transfer. Primary data extraction included specific enumerated complications of fat necrosis, infection, induration/calcification, oil cysts and haematoma. Acute complications were classified as being less than four weeks from the time of surgery, whilst delayed complications were over four weeks after fat transfer. Secondary data outcomes extracted were geographic location of article, article type and duration of study, mean age of patient, follow-up time, operating time and fat processing method. Before analysis, the heterogeneity between studies was described using the Cochran's Q test [17]. Cochran's Q test indicated the presence of heterogeneity; hence, random-effects models were used throughout. Statistical analysis was performed using Stats Direct (StatsDirect Ltd, Cheshire, UK). Statistical significance threshold was determined as p <0.05. Data, where possible, will be described as mean or median with range values. 

Results 

**Figure 1:**
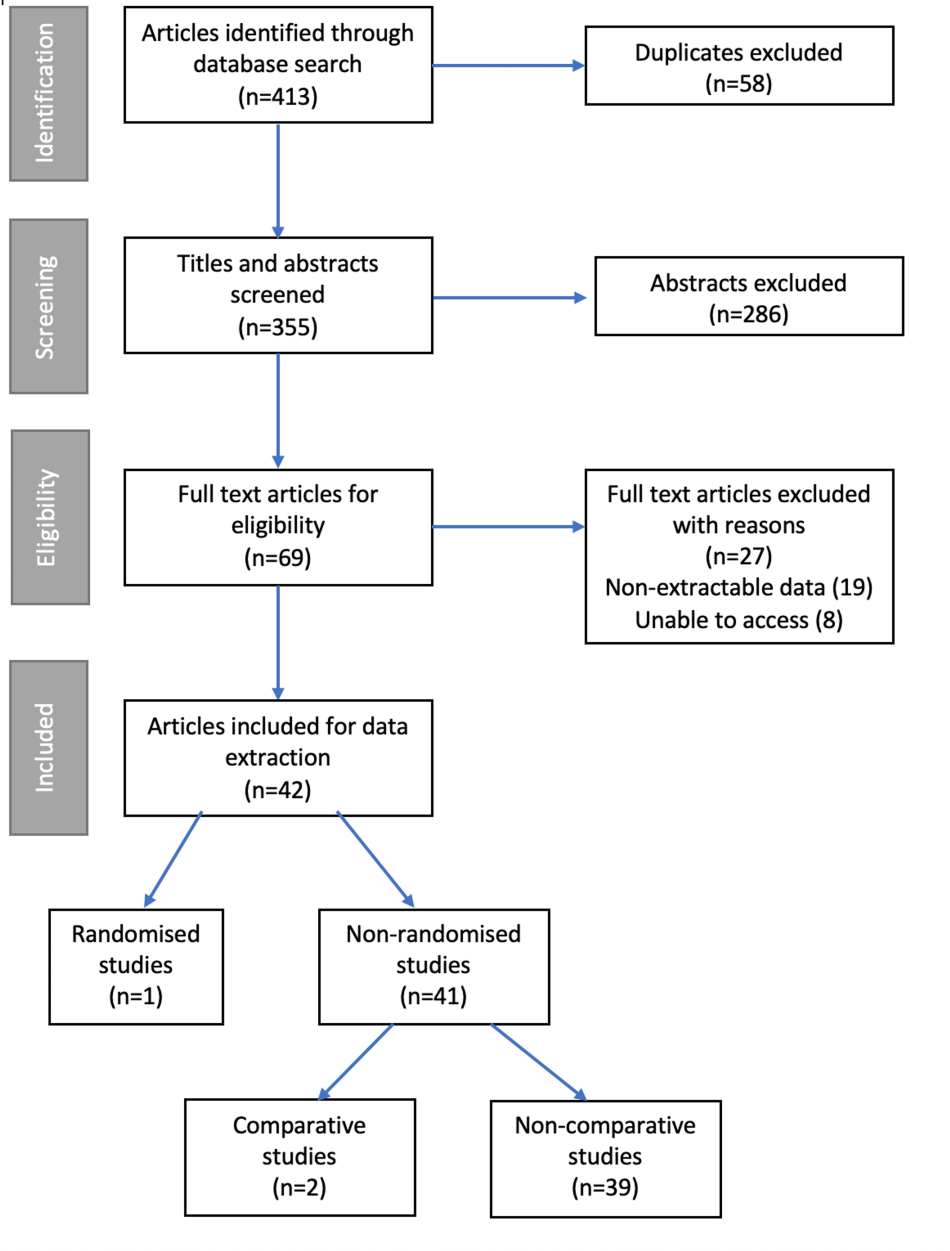
Flow diagram showing the search algorithm on application of the inclusion and exclusion criteria.

On initial search, 413 articles were identified. After application of inclusion and exclusion criteria and removal of duplicates, 42 articles were included in the final analysis [14,24-65]. Of these articles, one was a randomised control trial (RCT) [39] and 41 were non-randomised. Of these 42 studies, two were comparative and 39 were non-comparative. The RCT Detsky score was 18 (considered high quality) with a non-randomised MINORS mean of 5.8. No non-randomised studies were considered to be of high quality. Figure 1 describes the selection algorithm. The agreement between researchers selecting papers revealed a kappa of 0.68.

A total of 6268 patients were included. Europe contributed 18 studies to this review (n=2524), with 14 from Asia (n=2372), eight from North America (n=962) and two from South America (n=410). The mean length of studies was 4.65 years (1-12 years). Article types were retrospective cohort (n=33), prospective cohort (n=8) and randomised control trial (n=1). The mean age of patients was 38.1 years (range 5.5-65 years). The mean follow-up after the first operation was 25.9 months (range 4-78 months). Mean operating time for fat transfer was 60.1 minutes (range 25-100 minutes). 

A wide range of surgical techniques and management protocols was described. Infiltration solution was used in 24 articles, with nine articles using Klein’s solution without specification of content (n=973). The median diameter of the harvest cannula was 3 mm (range 1.8-4 mm). The abdomen was the most common site for fat harvest (12 studies), followed by the thighs (11 studies), flanks (eight studies) and buttocks (four studies). 

Fat processing using centrifugation was used in 29 studies, whilst six studies used a gravity method using gauze. Of the 29 studies using centrifugation, the median parameters used were 3000 revolutions per minute (range 360-3000) for 3 minutes. Nineteen out of 26 articles used the Coleman centrifugation protocol of 3000 revolutions per minute for 3 minutes [2]. The mean total volume of fat transferred was 178.8 ml (range 2.1-1020 ml).

**Figure 2:**
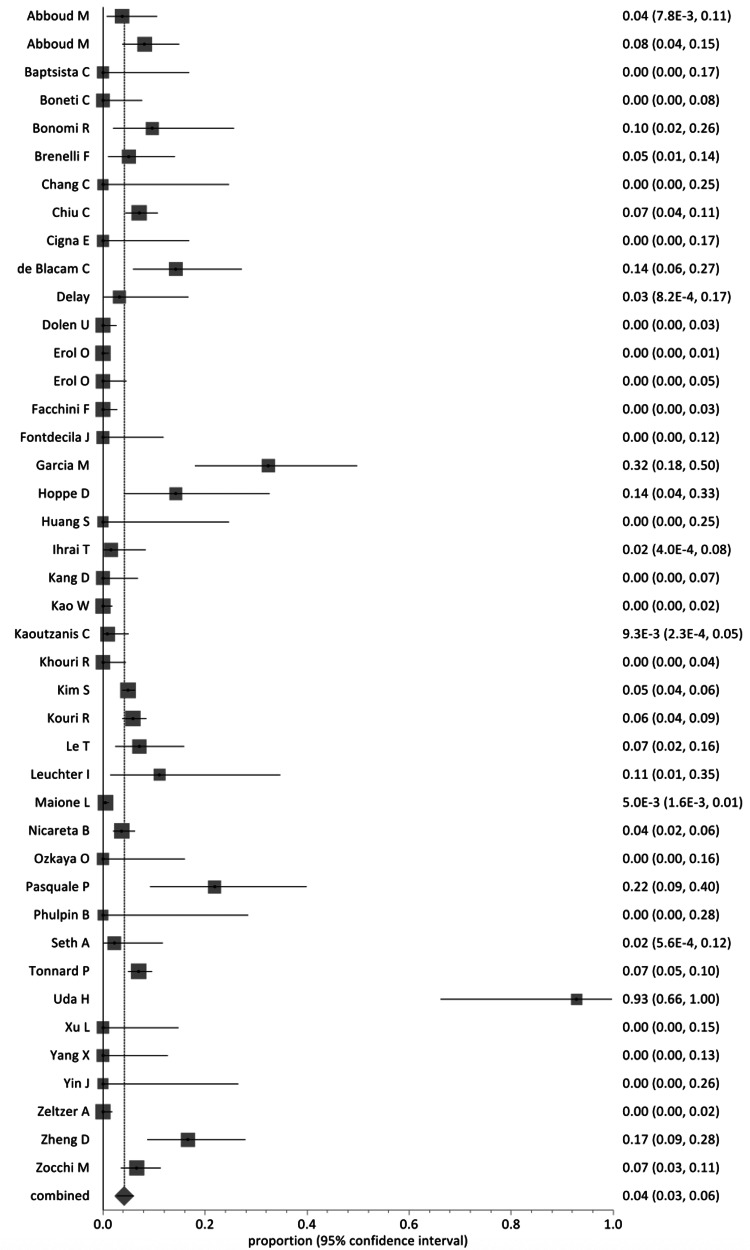
Forest plot showing the proportion of reported complications after fat transfer (42 studies, 6268 patients). Individual study results are given within the figure. A summary statistic of the random-effects model shows the total incidence of complications as 4.2% (95% CI=2.7-6.0). CI=confidence interval. Source: [14,24-64].

**Figure 3:**
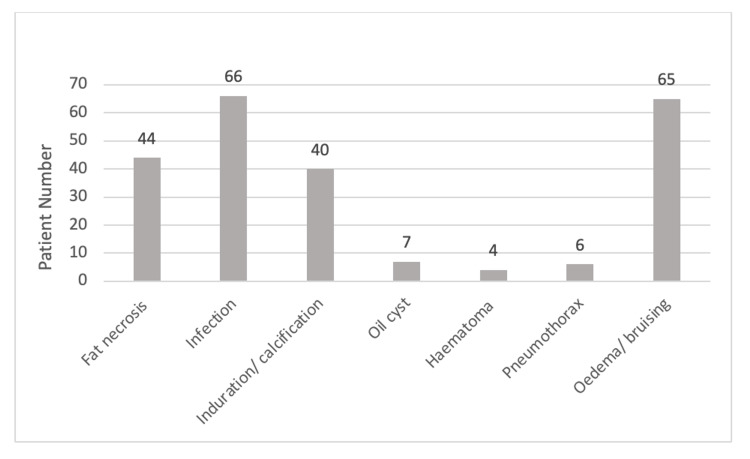
Bar chart showing the number of patients for reported complications of fat transfer. Specific enumerated complications included fat necrosis (n=44, 0.7%), infection (n=66, 1%), induration and calcification (n=40, 0.6%), oil cysts (n=7, 0.1%) and haematoma (n=4, 0.06%). Post-procedure pneumothorax was demonstrated in six patients (0.1%). The remaining patients (n=65) had chronic oedema/bruising.

**Figure 4:**
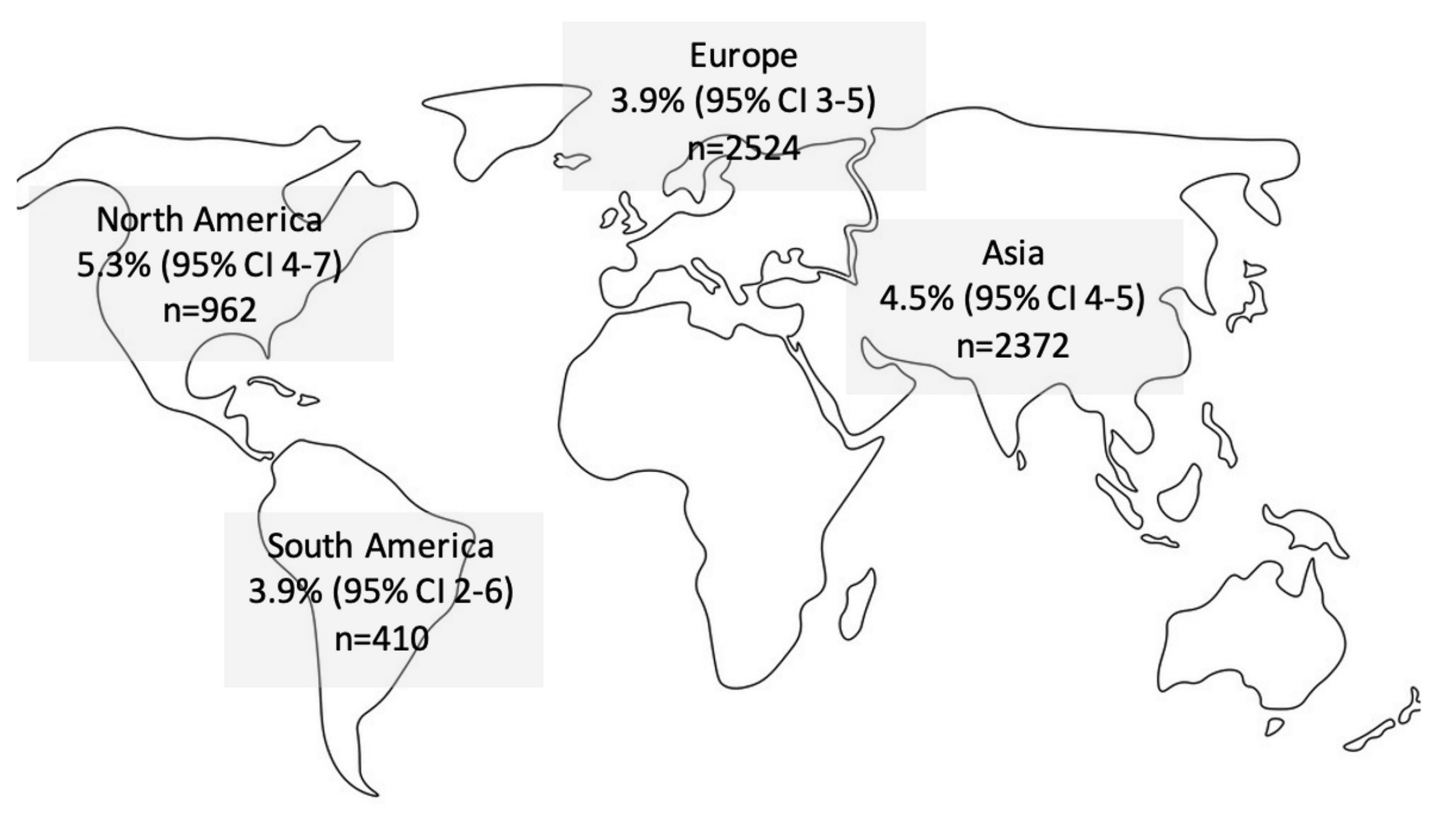
Incidence of complications by continent. n=total number of patients in a given geographical area and the incidence (percentage) of complications; CI=confidence interval. Source: https://freesvg.org/map-of-the-world-outline.

The crude incidence of complications was 4.2% (Figure 2). Specific enumerated complications (Figure 3) included fat necrosis (n=44, 0.7%), infection (n=66, 1%), induration and calcification (n=40, 0.6%), oil cysts (n=7, 0.1%) and haematoma (n=4, 0.06%). Post-procedure pneumothorax was demonstrated in six patients (0.1%). The remaining patients (n=65) had chronic oedema/bruising. By continent (Figure 4), Europe had a pooled proportion of complication incidence of 3.9% (95% CI: 3-5%), Asia 4.5% (95% CI: 4-5%), North America 5.3% (95% CI: 4-7%) and South America 3.9% (95% CI: 2-6%). 

**Figure 5:**
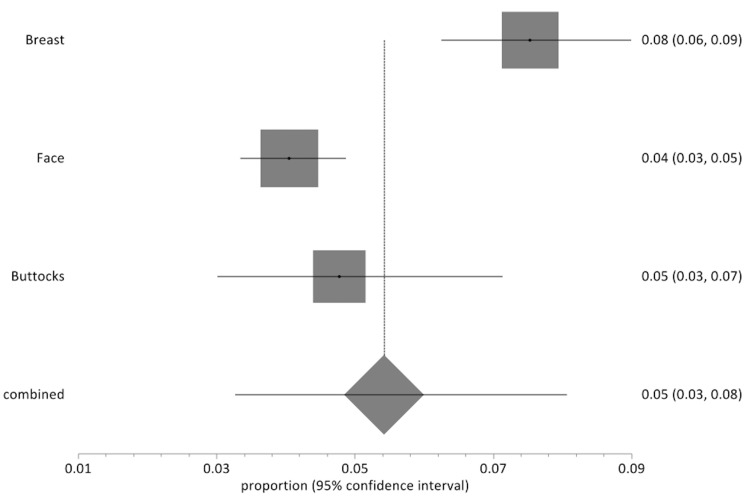
Forest plot showing the proportion of reported complications after fat transfer for each recipient body site. Fat transfer to the breast had an overall complication rate of 7.5% (95% CI: 6-9, n=112/1487 patients), the face 4.0% (95% CI: 3-5, n=109/2691 patients), buttocks 4.8% (95% CI: 3-7, n=22/461 patients) and palate 3.1% (95% CI: 3-4, n=2/64 patients). CI=confidence interval.

Fat transfer to the breast had an overall complication rate of 7.5% (95% CI: 6-9, n=112/1487 patients), the face 4.0% (95% CI: 3-5, n=109/2691 patients), buttocks 4.8% (95% CI: 3-7, n=22/461 patients) and palate 3.1% (95% CI: 3-4, n=2/64 patients). Figure 5 illustrates the random-effects model for complications of each body site. Fat transfer to the face was a significant contributor to our study, with 2736 patients from 14 papers. The overall complication rate of autologous fat transfer to the face was 4.0% (109 out of 2736 patients). Fat transfer to the buttocks contributed to 461 patients from two papers, with a total complication rate of 4.7% (22 out of 461 patients). Fat transfer to the palate contributed to 64, respectively, from one paper, with a total complication rate of 3.1% (two out of 64 patients).

Discussion

Kim et al. [49] described the largest study in this literature review with n=1261 patients. The authors conducted a retrospective chart review of all patients in a four-year time period who underwent full-face fat injections for face augmentation using up to three injections. The first injection was using fresh fat, with the second and third using frozen fat [49]. The authors described complications in 62 patients, giving an overall complication rate of 4.9%. This was higher than the overall crude complication rate of 4.2% and also higher than the overall face fat transfer complication rate of 4.0% (95% CI: 3-5, n=109/2691 patients). Kim et al. [49] described acne as being the most common complication after the first face fat injection, calcification after the second fat injection, and calcification with fibrosis after the third fat injection. The authors also found that acne showed increased frequency following the use of fresh fat, whereas the use of frozen fat resulted in a higher frequency of calcification and fibrosis [49]. The authors also described a statistically higher complication rate for the second and third fat injections with frozen fat, after the first injection with fresh fat [49]. This may explain their higher complication rate compared to the average, though the mechanisms of this are not fully understood. The incidence of infection after fat transfer was a comparable 1.1% in their paper [49], with an overall infection incidence of 1% in all papers. Phulpin et al. [56] described the smallest article in this review by patient number. The authors described a retrospective case series of 11 radiotherapy patients who underwent head and neck fat grafting after abdominal fat harvest. The authors described no complications during their 39.9 (range 2-88)-month follow-up [56].

Spear et al. described an 8.5% incidence of infection and fat necrosis in 37 patients with reconstructed breasts [19]. The authors examined 68 reconstructed breasts with a total of 111 discrete fat injections. More than one fat injection was received by 71.4% of breasts [19]. This may account for the higher incidence of infection and fat necrosis compared to our study (4.2%). In addition, Spear et al. [19] observed that patients had greater complications with increased volumes of fat grafting (184 ml versus 99 ml, p=0.0115). Our study had a mean breast transferred volume of 263.1 ml, considerably higher than the 184 ml of the Spear et al. cohort, and with a lower complication incidence. The higher rate of infection and fat necrosis observed in the Spear et al. [19] study may be due to the study’s small cohort of 37 breast patients, compared to our study of 2894 breast patients. 

Largo et al. [11] performed a literature review of autologous fat grafting to healthy breast tissue, including 36 articles and 1453 patients after application of their inclusion and exclusion criteria. Their study states an overall complication rate of 15.6% with a mean follow-up of 16.3 months [11] compared with 8.0% in our study. Largo et al. had a higher complication rate that can be explained by the authors including dysaesthesia, lymphadenopathy and unsatisfactory cosmesis into their cohort as complications. Their overall rate of infection was low at 0.7% (10 out of 1453 patients) [11] compared to our study of 2.2% (32 out of 1487 patients). 

Uda et al. [59] demonstrated the highest complication rate in our study, with 13 out of 14 patients suffering complications (11 patients with dermatitis and two patients with fat necrosis). Fat transfer was performed in conjunction with the Brava device (a vacuum-assisted method of breast augmentation) [59]. This high incidence of complications may be explained by all patients in this study undergoing radiotherapy, impairing the quality of the skin [59]. Additionally, the Brava device may introduce a bias into this study, as complications may be attributed to the device, rather than fat transfer. 

This study was limited by a data set that was heterogeneous, and this is a limitation of this review. A paucity of homogeneous patient comorbidity data made it challenging to draw adequate conclusions about complications linked to patient health. A large variety of harvest and fat-processing techniques were used from a wide range of donor sites (e.g., lateral thigh, abdomen, flank and gluteal). Infiltration solution used before fat harvest was also varied in terms of the volume used and constituents (saline 0.9% vs Ringer’s lactate, concentration of adrenaline and choice of local anaesthetic). Fat processing was also not uniform, ranging from using a centrifuge to purified fat, mesh strainers and other filters. As such, sub-group analysis was challenging to compare ‘like with like’ datasets. 

## Conclusions

We would recommend standardisation in complication recording and classification. Given the variation in harvest, processing and grafting methodology, we would also recommend multi-centre randomised control trials of surgical procedure to optimise fat grafting and limit complications in the future.

To our knowledge, we present the largest literature review of autologous fat transfer in the literature. This study is unique in its scope and size and can be used for counselling patients in the pre-operative setting and providing a benchmark for surgeons to assess their practice.
